# The role of circulating thrombospondin-1 in patients with precapillary pulmonary hypertension

**DOI:** 10.1186/s12931-016-0412-x

**Published:** 2016-07-30

**Authors:** Ralf Kaiser, Christian Frantz, Robert Bals, Heinrike Wilkens

**Affiliations:** 1 Department of Internal Medicine V Pulmonology, Allergology, Respiratory Intensive Care Medicine, Saarland University, Kirrberger Strasse, D-66424 Homburg/Saar, Germany; 2 Department of Pulmonology, Hôpitaux Robert Schuman – Zithaklinik, 38-40 Rue Sainte Zithe, L-2763 Luxembourg, Luxembourg

## Abstract

**Background:**

The vasoconstrictive protein TSP-1 is released from endothelial cells upon increased shear stress and hypoxia. Both conditions are prevalent in pulmonary hypertension (PH). TSP-1 damages the local microcirculation by disrupting pathways, which are essential for specific medical therapeutics. Furthermore, TSP-1 induces excessive fibrosis and smooth muscle proliferation - a common finding in advanced PH - via TGF-ß and might promote disease progression. The prognostic impact of circulating TSP-1, influence on hemodynamic parameters and interaction with other biomarkers in patients with PH is incompletely understood.

This study examines prospectively circulating TSP-1 in association with hemodynamic parameters, clinical variables and mortality.

**Methods:**

Circulating TSP-1 was measured prospectively in 93 patients with precapillary PH undergoing right heart catheterization and in 19 subjects without PH. TSP-1 levels were determined by ELISA and examined in the context of hemodynamic variables. For evaluation of survival, patients were monitored for adverse events on a 3-monthly basis and contacted at the end of the study after 5 years. In addition, levels of big-endothelin and humoral cofactors of TSP-1 release were measured.

**Results:**

Patients with PH had significantly increased TSP-1 levels compared to controls without PH (1114 ± 136 ng/mL vs. 82.1 ± 15.8 ng/mL, *p* < 0.05). Levels were correlated with mean pulmonary artery pressure (PAPm, r = −0.58, *p* < 0.001) and pulmonary vascular resistance (PVR, r = 0.33, *p* = 0.002). Survivors had lower TSP-levels as non-survivors and all cause mortality associated with TSP-1 plasma levels above 2051 ng/mL (*p* = 0.0002, HR 1.49).

**Conclusions:**

High plasma levels of TSP-1 are associated with increased PAPm, increased PVR and decreased survival. Due to its interaction with therapeutic pathways, studies are warranted to clarify the impact of TSP-1 on of specific medications for PH.

**Electronic supplementary material:**

The online version of this article (doi:10.1186/s12931-016-0412-x) contains supplementary material, which is available to authorized users.

## Background

Thrombospondin-1 (TSP-1) is a matricellular protein, which is involved in various processes of inflammation, neovascularization, hypoxia and remodeling [1–3]. A major source of TSP-1 are the alpha-granules of platelets [4]. Nevertheless, it is expressed in numerous cell types like macrophages, endothelial cells, vascular smooth muscle cells and cardiomyocytes [5–7]. Its production increases dramatically under conditions of hypoxia, altered shear stress or exposure to growth factors including platelet derived growth factor and basic fibroblast growth factor [8]. These conditions are all present in pulmonary hypertension. In vitro and in vivo studies revealed three mechanisms of action of TSP-1. First, binding to CD47 disrupts the nitric oxide pathway and vascular endothelial growth factor pathways based on the inhibition of soluble guanylyl cyclase and protein kinase G in vascular smooth muscle cells [9]. Second, in higher concentrations TSP-1 binds and activates CD36, thereby decreasing intracellular cGMP-levels due to interaction with cellular uptake of myristic acid and decreased eNOS-dependent synthesis of nitric oxide [10]. The net result of TSP-1 binding to these receptors is induction of apoptosis and abortion of VEGF and nitric oxide signaling [11–13]. Finally, TSP-1 is able to exert its biological effects by an additional mechanism involving the activation of transforming growth factor ß (TGF-ß) leading to excessive fibrosis [14]. Therefore, TSP-1 is able to diminish the effects of specific therapeutics of PH and promote disease progression of PH.

In this study we investigated the possible associations of circulating TSP-1 with clinical as well as hemodynamic variables and survival in PH. TSP-1 levels were compared between a control group without PH, patients with pulmonary arterial hypertension (PAH), lung disease associated PH (LD) and chronic thromboembolic PH (CTEPH). Furthermore, humoral cofactors of TSP-1 release were measured and analyzed.

## Methods

### Participants

Between June 2007 and December 2010, we enrolled prospectively 19 control subjects and 93 patients prospectively from the outpatient clinic for PH at the Department of Pulmonology of the Saarland University Hospital (Homburg / Saar, Germany). The control group consisted of subjects without evidence of any internal disease. In nine out of nineteen controls a right heart catheterization was performed for exclusion of pulmonary hypertension while in nine persons without any medical history of chronic disease or respiratory symptoms, PH was considered absent. Cardiopulmonary function tests including echocardiography and right heart cathererization were within normal range. The patients had incident and prevalent diagnosis of PH and had diagnostic workup according to current guidelines. Cardiovascular diseases aside from PH were excluded prior to inclusion into the study. Diagnosis of PH was based on a mean pulmonary artery pressure above 25 mmHg (PAPm) and a pulmonary artery occlusion pressure (PAOP) below 15 mmHg. Cardiac output was determined by thermodilution method. All patients received pulmonary ventilation/perfusion scintigraphy to diagnose CTEPH. Patients with suspected CTEPH had confirmatory pulmonary angiography. The inclusion into the study was irrespective of operability and plasma samples were taken before planned pulmonary end-arterectomy. Both incident as well as prevalent diagnosis of PH was included. Patients were at least two months clinically and hemodynamically stable before enrollment as indicated by unchanged functional class, lack of hospitalization and stable medication.

The study complies with the declaration of Helsinki and was approved by the local ethics committee (Ethikkommission der Ärztekammer des Saarlandes, Nr. 153/11). Written informed consent was obtained from each patient.

### Measurements

Blood was drawn during right heart catheterization as initial work-up or follow-up. For platelet stabilization, blood samples were collected into tubes containing 1 mL volume of citrate 109 mmol/l, theophylline 15 mmol/l, adenosine 3.7 mmol/l, dipyridamole 0.198 mmol/l. Plasma was separated by immediate centrifugation (2700xg at 4 °C, 20 min) and stored at −80 °C for batch analysis. The local clinical laboratory determined routine parameters.

TSP-1 levels were measured by ELISA in duplicates according to the manufacturers instructions (Quantikine human TSP-1 ELISA, R&D, Minneapolis, USA). Optical density was determined using a Tecan Spectra III reader set to 450 nm and 620 nm reference wavelength. The TSP-1 assay had a minimal detection limit of 0.355 ng/mL. The within run repeatability coefficient of variation was 5.1 %. Inter-assay repeatability was calculated with 11.3 % resulting in an overall variability of 8.2 %.

PGDF-β platelet poor plasma levels were measured by the Quantikine Human PDGF-β Immunoassay (R&D Systems, Minneapolis, USA, Catalog Number DBB00). Big-Endothelin ELISA was obtained from the Biomedica Group (Vienna, Austria, Catalog Number BI-20082), SDF1a and PF-4 ELISA from Ray Biotech, Inc. (Catalog Number ELH-SDF1alpha-001, ELH-PF4-001). Measurements were performed as indicated by the manufacturer.

### Statistics

Continuous variables were tested for normality and variance homogeneity by Shapiro-Wilk test and Hartley´s F_max_ test respectively. Non-normally distributed variables are expressed as median (range), normally distributed variables as mean ± SD unless indicated otherwise. Categorical variables are expressed as numbers (percentage). Outliers were defined outside a three-SD interval from mean of the whole dataset. Differences between groups were tested by ANOVA with post-hoc analysis (Dunnett T3 correction) for normally distributed and Mann–Whitney-*U*-test for non-normally distributed variables. Comparison of categorical variables was performed by Chi-square-test.

Regression analysis was studied for associations of continuous variables. For bivariate regression a non-parametric model was chosen with locally linear curve fitting of the dataset.

Kaplan-Meyer-Analysis for death was performed with an optimized cut-off of TSP-1 by minimized Matthews coefficient. Hazard ratios were calculated using a cox proportional hazard model (efron method). A p-value of less than 0.05 (two-tailed) was considered statistically significant in all tests.

Data storage and processing, calculations, as well as statistical computations were performed by R-Project version 3.2.0.

## Results

### Study population

The characteristics of the study population are detailed in Table 1 (*n* = 112). 93 patients and 19 control subjects were enrolled. The mean age was slightly higher in patients compared to control subjects (59 years vs. 47 years). 61 % of the population was female. Standardized WHO functional class was provided by the treating physician in our center. In the control group, workup including laboratory examinations, cardiopulmonary exercise test (CPET), catheter techniques (*n* = 9) and pulmonary function testing, excluded physical illnesses beyond reasonable doubt. The PAH group consisted of 35 patients with idiopathic PAH, 15 patients with associated PAH (nine connective tissue disease, three Eisenmenger, two sclerodermia, one HIV) and two patients with familial PAH. Three patients had drug induced PAH. Patients with LD had idiopathic pulmonary fibrosis (*n* = 11), COPD (*n* = 6), one patient with exogenic allergic alveolitis, and four patients with obstructive sleep apnea and fixed PH under guideline therapy. 16 patients had chronic thromboembolic pulmonary hypertension, 38 patients (33.9 %) were incident PH and treatment naïve upon inclusion.

**Table 1:** Basic characteristics of control group and patients grouped by DanaPoint classification

	Control	All PH	PAH	LD	CTEPH
n	19	93 (100)	55 (59.1)	22 (23.7)	16 (17.2)
Male	7	37	19	15	3
Age [y]	47.2 ± 14.9	59.3 ± 15.8*	55.1 ± 16.9^(^*^)^	66.9 ± 13.0*,**	63.0 ± 9.9*,^(^**^)^
Height [cm]	169.2 ± 10.1	167.1 ± 9.8	165.6 ± 8.9	170.1 ± 11.9	168.4 ± 9.0
Weight [kg]	71.7 ± 17.3	81.0 ± 20.0^(^*^)^	81.6 ± 20.2	82.2 ± 23.7	76.8 ± 11.9
BSA [sqm]	1.81 ± 0.23	1.89 ± 0.24	1.88 ± 0.23	1.92 ± 0.30	1.87 ± 0.17
FC II-III	0	74	44	17	13
6MWD	441 ± 68.4	328 ± 142*	353 ± 110	243 ± 155*,**	347 ± 192^(^***^)^
PDEI	0	43 (46.2)	28 (51.0)	11 (50.0)	4 (25.0)
ERA	0	39 (41.9)	29 (52.7)	2 (9.1)	8 (50.0)
PC	0	14 (15.1)	12 (21.8)	0	12 (12.5)
Other	0	6 (9.5)	4 (10.8)	2 (11.8)	0
No drug	16 (83.4)	19 (20.4)	6 (10.9)	8 (36.4)	5 (31.3)
1 Drug	3 (16.6)	45 (48.4)	26 (47.3)	12 (54.5)	7 (43.7)
2 Drugs	0	25 (26.9)	19 (34.5)	2 (9.1)	4 (25.0)
≥3 Drugs	0	4 (4.3)	4 (7.3)	0	0

*BSA* body surface area determined by DuBois formula, *FC* modified NYHA functional class, *6MWD* 6 min walking distance. Specific drug therapy: *CCB* calcium channel blocker, *PDEI* phospho-diesterase inhibitor, *ERA* endothelin receptor antagonist, *PC* prostacyclin (inhaled/subcutaneous)

*p < 0.05 vs. Control, **p < 0.05 vs. PAH, ***p < 0.05 vs. LD, marks in brackets signify statistical trends (0.05 < *p* < 0.01), absolute case numbers (% of cases in group)

Six minute walking distance (6MWD) was determined according to guidelines. 79 % of patients were in functional class II or III. Six minute walking distance in control patients was 441 ± 68.4 m and significantly lower in patients with PH patients (328 ± 142 m; *p* < 0.05). Lowest values for 6MWD were observed in patients with lung disease (243 ± 155 m), while patients with PAH had a walking distance of 353 ± 111.3 m.

Current medication is summarized in Table 1. 20.4 % of patients received no specific therapy at enrollment and none of the control group received specific drugs. 75.3 % of patients received one or two specific drugs, mostly endothelin receptor antagonists (ERA) or phosphodiesterase inhibitors (PDEI). Only 4.3 % were on triple therapy.

### Circulating TSP-1 and biomarker levels

**Fig. 1:**
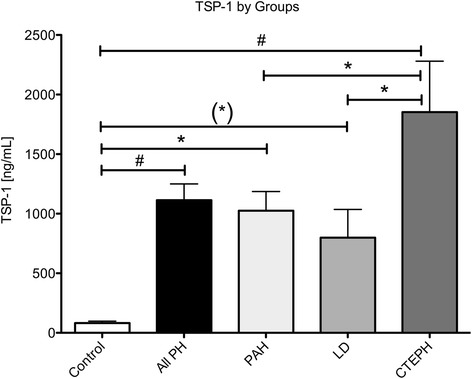
Data represents mean ± SEM of circulating TSP-1 levels by groups according to Nice classification

**Fig. 2:**
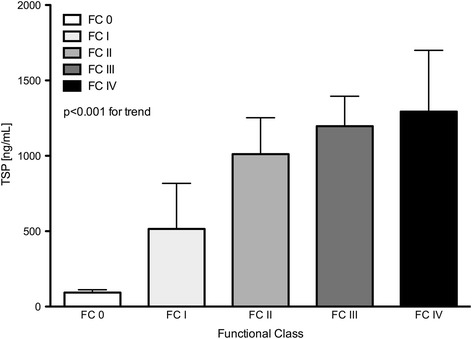
Data represents mean ± SEM of circulating TSP-1 levels by WHO functional class

Patients with PH had significantly higher levels of circulating TSP-1 compared to healthy controls (Table 2, Fig. 1). The subgroup analysis showed the lowest values within the lung disease associated PH group (LD). Patients with PAH or CTEPH demonstrated a 12.5 times and 22.6 times increased concentration of circulating TSP-1 compared to controls, respectively. Among all tested subjects, TSP-1 increased with WHO functional class (Fig. 2, *p* < 0.001). Comparing incident with prevalent PH, no significant difference in TSP-1 levels were found (752 vs. 1053 ng/mL, *p* = 0.2) and no differences between hemodynamic parameters were apparent. No influence of either gender, age or kidney function was statistically significant.

**Table 2:** Circulating biomarkers grouped according to DanaPoint classification

	Control	All PH	PAH	LD	CTEPH
TSP-1 [ng/mL]	82.1 ± 15.8	1114* ±136	1025* ±161	799* ± 237	1852*,**,*** ±428
bigET [pg/mL]	1.22 ± 0.34	2.96* ±0.34	3.05* ±0.48	2.29 ± 0.53	4.06* ±0.75
PDGF-ββ [pg/mL]	152 ± 11	784* ±161	905 ± 253	652* ±145	382 ± 173
PF4 [pg/mL]	2.12 ± 0.25	3.05^(^*^)^ ±0.29	2.81 ± 0.30	3.60 ± 0.72	2.95 ± 0.86
SDF-1α [pg/mL]	71.6 ± 8.7	124.5* ±14.2	128 ± 21.4	121 ± 17.1	116 ± 46.1
NT-proBNP [pg/mL]	128.0 ± 49	1212* ±214	1070* ±213	1529 ± 662	1296* ±495
Creatinine [mg/dL]	0.77 ± 0.22	1.01 ± 0.32*	1.06 ± 0.32*	0.95 ± 0.26	0.94 ± 0.35
Uric acid [mg/dL]	5.09 ± 1.41	7.56 ± 2.46*	7.20 ± 2.13*	7.70 ± 2.12*	8.93 ± 4.01*
Hb [g/dL]	14.2 ± 1.3	14.2 ± 2.4	14.6 ± 2.5	13.9 ± 2.3	13.5 ± 1.9
LDH [U/L]	199.9 ± 66.0	285.6 ± 91.0*	277.6 ± 62.0*	279.5 ± 70.9*	324.2 ± 173.2*
Bilirubin [mg/dL]	0.55 ± 0.20	0.80 ± 0.50	0.78 ± 0.47	0.78 ± 0.39	0.88 ± 0.75

*TSP-1* thrombospondin 1, *bigET* big Endothelin, *PDGF-ββ* platelet derived growth factor ββ, *PF4* platelet factor 4, *SDF-1α* stroma derived factor 1α, *NT-proBNP* N-terminal pro-brain natriuretic peptide

Data is expressed as mean ± SD, *p < 0.05 vs. Control, **p < 0.05 vs. PAH, ***p < 0.05 vs. LD, marks in brackets signify statistical trends (0.05 < *p* < 0.01)

Patients with PDEI had lower TSP-1 levels compared to patients without PDEI (1234 vs. 516 ng/mL, *p* = 0.02). In contrast, ERA therapy had no effect on TSP-1 levels (806 vs. 1188 ng/ml, *p* = 0.1), while patients on PC-therapy had higher TSP-1 levels (823 vs. 1762 ng/ml, *p* = 0.01).

Circulating levels of PDGF-β - a potent inductor of TSP-1 - were elevated in PH compared to controls, with PAH showing the highest levels followed by lung disease associated PH. Levels of patients with CTEPH and PAH were elevated but not statistically significant (Table 2).

Platelet factor 4 (PF4) is an established marker of platelet activation and was determined in the same plasma samples. Despite a trend to increased levels in the PH group, none of the subgroups reached statistical significance (Table 2). No correlation of PF4 and TSP-1 was detected (rho = 0.252 with *p* = 0.471).

Among many biomarkers SDF-1α has been promoted to quantify endothelial shear stress. In patients with PH, SDF-1α was increased significantly, but the subgroup analysis produced no statistical significance for one of the types of PH (Table 2).

Big-endothelin showed significantly increased levels in both PAH and CTEPH patients, while levels in the group with LD did not reach statistical significance. Highest levels of big-endothelin were observed in CTEPH patients. NT-pro-BNP was statistically significantly elevated in PAH and CTEPH. The increased levels of NT-proBNP in the lung disease group were not statistically significant due to the high standard deviations (Table 2).

### TSP-1 and hemodynamic parameters

**Fig. 3:**
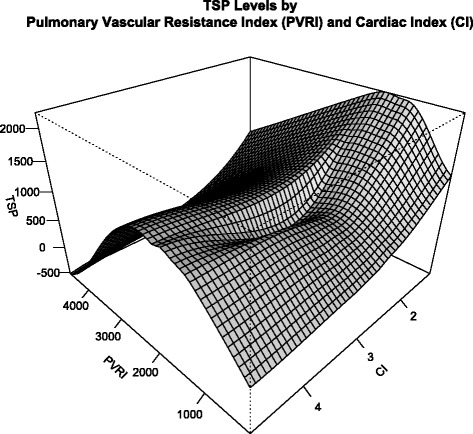
Locally linear non-parametric regression of circulating TSP-1 by PVRI and CI. The graph shows circulating TSP-1 levels based on a non-parametric regression model, which uses linear functions to fit the local data points. The model resembles a saddle form, suggesting a relative minimum of net TSP-1 production at normal CI and and increase of TSP-1 with both low and high CI. While TSP-1 increases with vascular resistance, extreme resistance might be due to loss of total vascular area and therefore reduction of TSP-1 producing cells. Hence, TSP-1 levels follow a reverse U-shape. This underlines the possible association of TSP-1 with shear stress at the pulmonary endothelium

The hemodynamic parameters are summarized in Table 3 as well as Additional file 1: Figure S1 and Additional file 2: Figure S2. Mean pulmonary artery pressure (PAPm) correlated positively with TSP-1 (rho = 0.579, *p* < 0.001), while cardiac output (CO) was negatively correlated (rho = −0.338, *p* = 0.002). Therefore, an increased TSP-1 was found in hemodynamically compromised patients. Consequently, PVR correlated positively with TSP-1 (rho = 0.331, *p* = 0.002). To demonstrate the complex interrelation of hemodynamic parameters with circulating TSP-1, a locally linear non-parametric regression was performed on TSP-1, PVR and cardiac index (Fig. 3). Here, an increase of TSP-1 can be seen as pulmonary resistance increases. In low resistance state, no TSP-1 is produced over a large range of cardiac output. In contrast, high resistance states produce a relative minimum at normal cardiac output.

**Table 3:** Summary of hemodynamic variables. All pressures are expressed in millimeter mercury

	Control	All PH	PAH	LD	CTEPH
PAPm	15.4 ± 2.1	54.2 ± 19.0*	57.0 ± 18.4*	44.9 ± 16.5*,**	59.1 ± 21.8*,***
PAPsys	23.6 ± 4.3	76.8 ± 23.4*	79.3 ± 22.0*	67.2 ± 24.2*,^(^**^)^	82.7 ± 25.0*,^(^***^)^
PAPdia	10.7 ± 2.5	31.3 ± 11.7*	34.3 ± 12.6*	26.6 ± 8.5*,**	26.6 ± 7.9*,**
PAOP	7.8 ± 2.3	9.8 ± 2.5*	9.7 ± 2.5	10.1 ± 2.7	9.8 ± 2.0
TPG	7.7 ± 2.1	44.5 ± 19.6*	47.5 ± 19.0*	34.8 ± 17.1*,**	49.2 ± 22.2*,***
CI	2.67 ± 0.6	2.46 ± 0.7	2.36 ± 0.7	2.67 ± 0.4	2.52 ± 1.0
PVRI	250 ± 125	1282 ± 690*	1411 ± 730*	923 ± 472*,**	1326 ± 661*

*PAPm* mean pulmonary artery pressure, *PAPsys* systolic pulmonary artery pressure, *PAPdia* diastolic pulmonary artery pressure, *PAOP* pulmonary artery occlusion pressure, *TPG* transpulmonary gradient, *CI* cardiac index [mL/min/sqm], *PVRI*, pulmonary vascular resistance index [dyn.s.cm^−5^.sqm]

Data is expressed as mean ± SD, *p < 0.05 vs. Control, **p < 0.05 vs. PAH, ***p < 0.05 vs. LD, marks in brackets signify statistical trends (0.05 < *p* < 0.01)

### TSP-1 and outcome

**Fig. 4:**
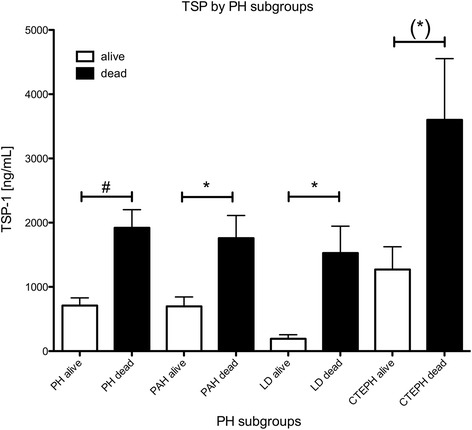
Data represents mean ± SEM of circulating TSP-1 levels by survivors and non-survivors in the various subtypes of PH

To determine the impact of circulating TSP-1 on prognosis, patients were followed by personal contact for five years, there was no loss to follow up. At the end of the study, 31 patients had died. Comparing survivors with non-survivors after five years, non-survivors had more than two-fold increased levels of circulating TSP-1 (Fig. 4). This was irrespective of the class of PH. Patients with CTEPH had an overall increased level of TSP-1 though.

**Fig. 5:**
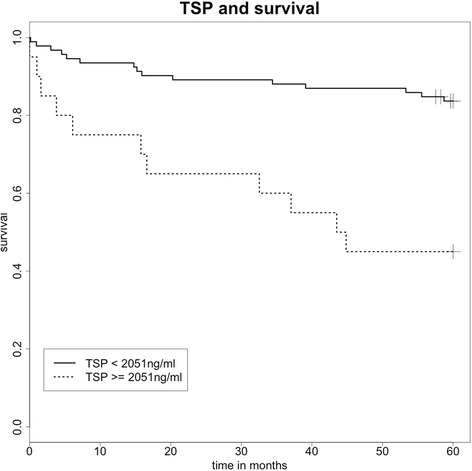
Kaplan-Meier-Analyses of survival for death at an optimized cut-off of >2051 ng/mL TSP-1 in plasma, *p* = 0.03

Using an optimized cut-off (minimal Matthew´s coefficient) of 2051 ng/mL, patients were grouped into high levels and low levels of circulating TSP-1. The Kaplan-Meier-analysis for survival showed a hazard ratio of 1.49 for cardiovascular death in case of TSP-1 levels above 2051 ng/mL (logrank test *p* = 0.002, Fig. 5).

## Discussion

The presented study showed, that levels of circulating TSP-1 differ significantly between patients with PH compared to subjects without PH. The subgroup analysis demonstrated the importance of the specific subtype of PH for the interpretation of this biomarker. Furthermore, associations of circulating TSP-1-levels and pulmonary hemodynamics were elaborated. And finally, the association of elevated TSP-1 with increased mortality became evident.

As Bauer and Isenberg showed, TSP-1 is abundantly expressed in lung tissue of patients with PH. In pulmonary endothelium, CD47 controls endothelial NO-synthase. Binding of TSP-1 uncouples eNOS via CD47 and promotes progress of hypoxic PH in animal models [15]. Furthermore, TSP-1 has been shown to deteriorate shear stress dependent vasodilation under hypoxic conditions and is induced by hypoxia in pulmonary vascular endothelium [16].

In this study we demonstrated a significant increase in plasma levels of TSP-1 in patients with various types of PH. While levels of TSP-1 varied in different subtypes of PH, the common denominator was their hemodynamic state. The univariate non-linear regression analysis showed a significant association of circulating TSP-1 with PVR and CO. This supports the initial hypothesis, that the increase of PVR increases shear stress resulting in a release of TSP-1 from pulmonary endothelial cells. However, hemodynamic parameters to determine shear stress in humans remain scarce. The extent of shear stress in a specific pulmonary vessel is influenced by both right ventricular function and properties of the vascular bed of the lung. PVR and cardiac output remain the best surrogate variables of standard right heart catheterization for estimation of shear stress.

On the other hand it has been shown, that TSP-1 production is minimal at optimal shear rates [17], explaining nonlinear U-shaped relation of shear stress and TSP-1 release. In this study we showed first clinical evidence for this complex relationship (Fig. 3), but had to omit possibly confounding factors (i.e. age, medication) due to limited data points.

Induction of TSP-1 in endothelial cells has been shown previously [18] and was confirmed for PAH [16]. Our data contrasts these findings, as an association of TSP-1 with mixed central venous oxygen saturation, which reflects peripheral oxygen depletion, could not be confirmed. Furthermore, the vascular endothelium might not be the only source of TSP-1 in PH, as increasing right ventricular load might release TSP-1 from cardiomyocytes as hemodynamics deteriorate. The release of TSP-1 from the myocardium especially due to ischemia-reperfusion injury has been shown previously [19] and might limit specificity of TSP-1 as a marker for diagnosis of PH.

Experimental data supported the hypothesis of TSP-1 interfering with the NO-pathway [20]. This abrogates soluble guanylate cyclase and protein kinase G as well as adenylate cyclase [21–23], resulting in direct pulmonary arterial vasoconstriction, disease progression [16] and disruption of key pharmacological targets of specific therapy.

Examination of drug regimes suggested lower TSP-1 levels on treatment with PDEIs, higher levels on PC-treatment and intermediate levels under ERA treatment. This observation might lead to the assumption, that treatment regime was influential on circulating TSP-1. On the other hand, PC therapy is an invasive therapeutic concept and often withheld until treatment goals are not sufficiently achieved by oral medications. Therefore, these observations might reflect the finding, that TSP-1 was elevated with deteriorating hemodynamics and increased functional class. The influence of medical therapy on TSP-1 levels could not be determined in this study and warrants longitudinal trials observing TSP-1 levels before and after initiation of new drug regimes.

As this study was designed as a cross-sectional observation, we included patients in different stages of disease. While some had newly diagnosed PH, others were in the final stage of their illness. Thus a non-matched comparison of TSP-1 levels between groups seems a serious limitation for this study. The comparison of incident and prevalent PH demonstrated slightly higher levels of TSP-1 in incident PH, statistical significance was not reached. While the groups had similar hemodynamics, the influence of medication, the course of disease and adaptive processes might lead to this finding. The reasons and possibly influence on the prognostic value of TSP-1 was beyond the scope of this cross-sectional pilot study.

Based on the data linking TSP-1 to hemodynamics and the possibility of its negative impact on pathophysiological mechanisms of PH, we sought to gain insight of the prognosis attached to TSP-1 as a biomarker. At the end of the study, survivor and non-survivors were compared and in all groups non-survivors had significantly higher levels of TSP-1 when entering the study (Fig. 4). CTEPH patients demonstrated relatively higher levels in both survivors and non-survivors compared to the other subgroups. It is therefore mandatory to exactly classify the type of PH before using TSP-1 as a potential biomarker.

Survival analysis in this study was based on follow-up in regular intervals up to five years. The patients had received standard therapy according to current guidelines. Cardiovascular mortality was chosen as the primary endpoint, but in this dataset it was identical with all cause mortality. We were able to show a significantly increased mortality in patients with high TSP-1 levels. This data is limited by the sample size of 93 patients and inclusion of further confounding factors or multivariate analysis with established predictors of mortality is obsolete. Nevertheless, the optimized cut-off at 2051 ng/mL yielded a highly significant difference between groups. Though, as discussed above, the circulating levels of TSP-1 might vary between subgroups and therefore a different cut-off can be expected especially in patients with CTEPH. Furthermore, serial measurements are needed to evaluate the true impact on survival and various clinical events in more complex frailty models.

Nevertheless, this data provides valuable information on the relation of TSP-1 with pulmonary hemodynamics, a clinical application of in vitro observations considering crucial pathways in PH and indicates an impact on the prognosis of a serious disease. These observations are the clinical implication of previous studies on the role of TSP-1 in experimental PH [15, 24].

The presented data might form the basis for evaluation of TSP-1 as an indicator for the choice of medication and might facilitate therapeutic decisions. TSP-1 interacts significantly with the NO pathway, thereby leading to uncoupling of specific therapeutic drugs [22]. Observational studies should evaluate TSP-1 as a marker for possible non-responders before initiation of therapy.

## Conclusions

In this observational study, we could show that TSP-1 levels are significantly elevated in PH. While the majority of studies focuses on strict selection of PH subgroups, this cross-sectional pilot study was able to compare various groups of precapillary PH. The association of TSP-1 levels with invasive hemodynamic parameters offers insight into the complex mechanisms of this disease. While current biomarkers focus on the consequences of PH such as myocardial damage, TSP-1 might monitor endothelial injury and activation at the very source of the pathology. In a prospective approach we were able to provide five-year survival data from patients with PH. Despite of the limited sample size, patients with elevated circulating TSP-1 levels demonstrated a lower survival rate. Therefore, prospective longitudinal studies are warranted to provide insight in the usability of TSP-1 to predict disease activity and progression and to confirm the usefulness of this marker in various clinical endpoints and decisions.

## Abbreviations

CI, cardiac index; CTEPH, chronic thrombembolic PH; LD, lung disease associated PH; NO, nitric oxide; PAH, pulmonary arterial hypertension; PAOP, pulmonary artery occlusion pressure; PAP, pulmonary artery pressure; PDGF, platelet derived growth factor; PF-4, platelet factor 4; PH, pulmonary hypertension; PVR/I, pulmonary vascular resistance/index; RAP, right atrial pressure; SAP, systemic artery pressure; SDF-1, stromal derived factor 1; TGF, tissue growth factor; TSP-1, Thrombospondin-1; VEGF, vascular endothelial growth factor

## Additional files

Additional file 1: Figure S1. Pulmonary artery pressures were determined invasively. Data is expressed as mean ± SD [mmHg]. Statistically significance is denoted in Table 2 for readability. (TIFF 384 kb) Additional file 2: Figure S2. Pulmonary vascular resistance index was derived from invasive measurements of PAOP, mean PAP and cardiac output by thermodilution method. Data is expressed as mean ± SD [dyn.s.cm^−5^]. Statistically significance is denoted in Table 2 for readability. (TIFF 270 kb)
