# Supplementary figures and images for: The role of circulating thrombospondin-1 in patients with precapillary pulmonary hypertension
Source: Respir Res. 2016 Jul 30;17:96. doi: 10.1186/s12931-016-0412-x (PMC4967340; doi:10.1186/s12931-016-0412-x)

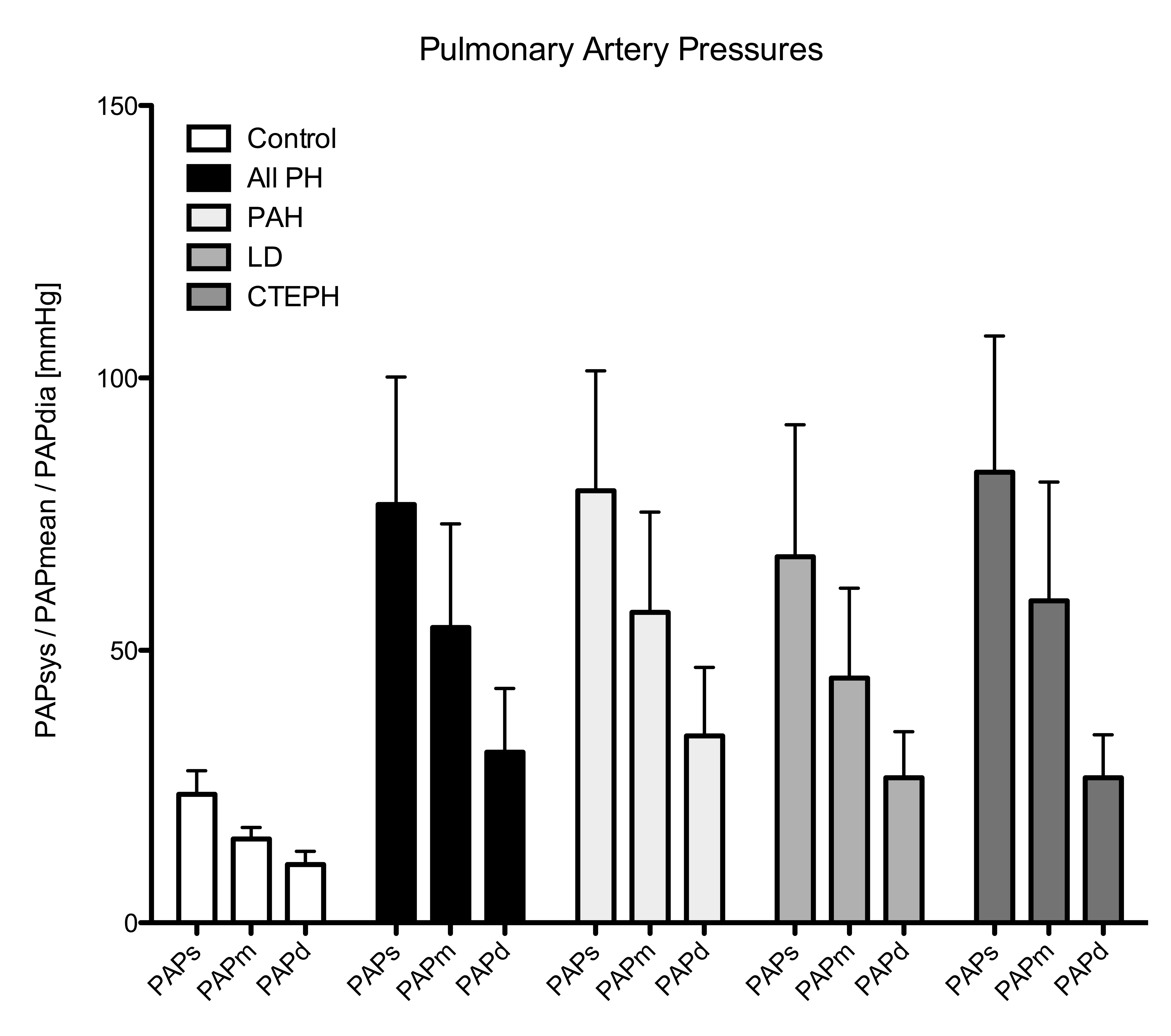

Supplement: Additional file 1: Figure S1. — Pulmonary artery pressures were determined invasively. Data is expressed as mean ± SD [mmHg]. Statistically significance is denoted in Table 2 for readability. (TIFF 384 kb) [file 12931_2016_412_MOESM1_ESM.tiff]

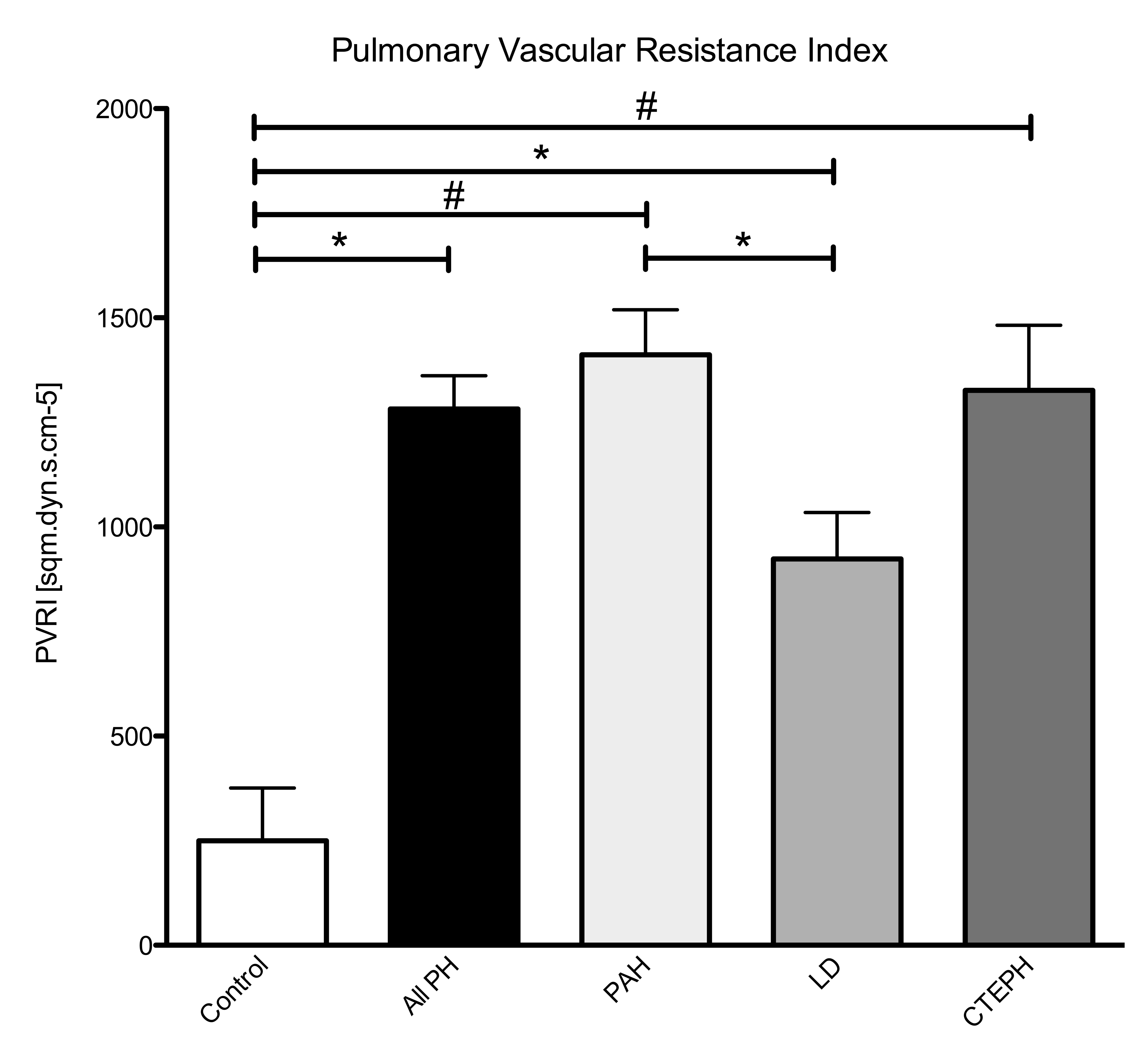

Supplement: Additional file 2: Figure S2. — Pulmonary vascular resistance index was derived from invasive measurements of PAOP, mean PAP and cardiac output by thermodilution method. Data is expressed as mean ± SD [dyn.s.cm−5]. Statistically significance is denoted in Table 2 for readability. (TIFF 270 kb) [file 12931_2016_412_MOESM2_ESM.tiff]
